# NAFLD and Physical Exercise: Ready, Steady, Go!

**DOI:** 10.3389/fnut.2021.734859

**Published:** 2021-10-05

**Authors:** Maja Cigrovski Berkovic, Ines Bilic-Curcic, Anna Mrzljak, Vjekoslav Cigrovski

**Affiliations:** ^1^Department of Kinesiological Anthropology and Methodology, Faculty of Kinesiology, University of Zagreb, Zagreb, Croatia; ^2^Department of Endocrinology, Diabetes, Metabolism and Clinical Pharmacology, Clinical Hospital Dubrava, Zagreb, Croatia; ^3^Department of Pharmacology, Faculty of Medicine, University of J. J. Strossmayer Osijek, Osijek, Croatia; ^4^Department of Endocrinology, Clinical Hospital Center Osijek, Osijek, Croatia; ^5^Department of Gastroenterology and Hepatology, University Hospital Center Zagreb, Zagreb, Croatia; ^6^School of Medicine, University of Zagreb, Zagreb, Croatia; ^7^Faculty of Kinesiology, University of Zagreb, Zagreb, Croatia

**Keywords:** non-alcoholic fatty liver disease, sedentary activities, physical activity, aerobic exercise, high-intensity interval training, strength training

## Abstract

Along with the increase in obesity and type 2 diabetes, the non-alcoholic fatty liver disease (NAFLD) incidence is escalating, thus becoming a leading cause of liver cirrhosis and a significant burden of liver-related outcomes. Since there is no pharmacotherapy available to address the NAFLD, the most effective solutions seem to be lifestyle changes centered on physical activity. Exercise could mediate its beneficial effects directly on the liver and indirectly via extrahepatic pathways, forming a dose-response relationship with NAFLD in terms of prevalence and disease severity. Health-enhancing physical activity (HEPA) levels are mainly needed to exert beneficial effects in obese subjects, while even a small amount of exercise can be beneficial for lean individuals to prevent NAFLD. This mini-review addresses three major points regarding physical activity and NAFLD: prevention, treatment, and extrahepatic benefits, offering recommendations on type and intensity of exercise in liver disease.

## Introduction

Non-alcoholic fatty liver disease (NAFLD) is an umbrella term inclosing a spectrum of clinical and pathological fatty liver disease entities which may lead to cirrhosis and hepatocellular carcinoma (HCC) (1). The prevalence of NALFD is increasing worldwide and is estimated at around 25% (2). However, the true prevalence of NALFD seems to be much higher, given the global rise of metabolic syndrome due to changes in eating habits and inclination toward a sedentary lifestyle.

Metabolic syndrome has become a growing morbidity cluster epidemic resulting in a sharp rise in obesity, type 2 diabetes mellitus (T2DM), hypertension, and dyslipidemia. Its liver manifestation—NAFLD has become the most common cause of the chronic liver disease (2). Moreover, the prevalence of NAFLD among patients with T2DM is even higher, 56%, while the overall prevalence of non-alcoholic steatohepatitis (NASH), a progressive form of NAFLD, reaches 37% (3). Finally, the incidence of NAFLD-related HCC, accompanied by life-threatening complications, is continuously increasing (4). Furthermore, lean individuals with NAFLD share the same severe histological phenotype as obese subjects and are associated with metabolic syndrome and an increased risk of all-cause mortality (5).

A recent meta-analysis assessing RCTs with dietary interventions but without any added physical activity tried to establish the effect of different dietary modifications on intrahepatic lipid content (IHL), liver fibrosis, and liver function in patients with NAFLD. The study showed Mediterranean diet without energy restriction leads to significant reduction of IHL. However, it is important to note that the diet without exercise did not lead to significant changes in liver enzymes, lipid profile, fasting glucose or insulin, or homeostatic assessment for insulin resistance. On the other hand, hypocaloric diet with foods high in unsaturated fatty acids significantly decreases ALT and AST, but its effects on steatosis remain to be established (6).

NAFLD development in obese and non-obese individuals is closely related to a sedentary lifestyle and a western diet (7). Physical activity, especially structured exercise, has been shown to improve hepatic steatosis and is the core treatment during the whole NAFLD disease spectrum. Physical activity has an essential role in weight reduction and maintenance, influences healthier body composition, reduces hepatic steatosis and NAFLD-associated cardiovascular and malignant burden (8). Importantly, modest weight gain in lean individuals has deleterious effects on metabolic disturbances primarily through increased visceral adipose tissue (9, 10). Bodyweight and waist circumference reduction achieved through lifestyle intervention are independent predictors of NAFLD resolution in lean patients (11).

## Physical Acitivty in Context of Liver Disease

Health-enhancing physical activity defined as either vigorous activity at least 3 days/week and accumulating at least 1,500 metabolic equivalents (METs)-minutes per week (MET-min/week) or seven or more days/week of any combination of walking, moderate, or vigorous activities accumulating at least 3,000 MET-min/week has been recently independently (after adjusted for confounders such as diet and obesity) associated with a lower risk of both NAFLD and lean NAFLD in the Asian population, while the risk of lean NAFLD was significantly lower even in minimally active lean individuals compared inactive lean individuals (adjusted OR, 0.8; 95% CI, 0.6–0.98) (12). Skeletal muscle as an endocrine organ secretes cytokines and myokines, through which, while working/contracting, it communicates with liver and adipose tissue, among others, and is involved in an anti-inflammatory response (13). In addition, physical activity (1,500 MET-min/week of vigorous or 3,000 MET-min/week of intermediate activity) significantly lowers the ALT levels and improves the hepatocellular injury in individuals with NAFLD (8). Although research regarding the effects of exercise on NAFLD is relatively recent, both experimental and clinical data support its importance, especially that of vigorous intensity, which effectively decreases intrahepatic lipid content and slows down the progression to NASH (14). In a small RCT including 24 individuals with biopsy-proven NASH, the benefit was seen from 12-week cycling and resistance training to decrease hepatic triglyceride content, plasma triglyceride levels, and visceral fat. However, no effects were reported concerning BMI, liver enzymes, or inflammation and fibrosis, suggesting weight managing strategies should be incorporated in NASH treatment (15). In patients with cirrhosis, exercise can acutely increase portal pressure, but it has positive health effects in the long term. Moreover, physical activity can improve the aerobic capacity, which is decreased in patients with advanced cirrhosis and adds to anyhow high mortality burden (16). In addition, by increasing skeletal muscle mass physical activity improves sarcopenia and reduces the risk of encephalopathy (17–20). Evidence regarding the effects of exercise on HCC risk is still scarce, but epidemiological studies suggest a lower risk in patients who regularly and vigorously exercise (21).

## Exercise and Nafld: What is Known on the Mechanism(S)

In many of the published studies, the effect of exercise on improvement of liver fat content was seen even in patients who did not achieve the weight loss therefore suggesting the direct effects on liver (22, 23). Although this direct relation is still largely elusive, the available evidence implies different metabolic and molecular pathways which are involved in the reduction of hepatic fat induced by exercise.

One of the most prominent and studied mechanisms is certainly related to insulin resistance (IR). Mechanistically, IR in peripheral tissues such as adipose tissue results in an incomplete suppression of lipase, leading to enhanced lipolysis and release of free fatty acids (FFAs), which are taken up by the liver (24). Therefore, an improvement in IR might reduce the FFA flux to the liver. Moreover, IR in skeletal muscle causes the glucose transport to the liver, which is the fuel for FFA *de novo* synthesis (25). The main transcription factor controlling liver fatty acid metabolism, sterol regulatory element-binding protein 1 (SREBP-1), which is elevated in the NASH can be decreased by either 12-week aerobic exercise of high intensity or resistance training through the increase of AMPK, leading to reduction of *de novo* lipogenesis in hepatocytes (26, 27). Moreover, exercise might also induce epigenetic mechanisms such as reduction of DNA hypermethylation which positively effects *de novo* lipogenesis (28).

In addition, exercise might also influence liver fatty acid metabolism by increasing expression of peroxisome proliferator-activated receptor-gama (PPAR-gama), in a similar way as the thiazolidinediones (29). Besides, animal models and small scale studies suggest exercise impacts liver mitochondrial function, and can influence inflammation through up-regulation of antioxidant enzymes and anti-inflammatory markers (24, 30).

## The Role of Physical Activity in the Nafld Prevention

Sitting for ≥3 h per day has been associated with increased all-cause mortality (relative risk 1.30; 95% CI 1.06–1.56), and sedentary behavior, in general, was reported higher in people predisposed to develop obesity T2DM, NAFLD, and metabolic syndrome (31). There is a strong association between increased hepatic triglyceride content and each hour spent sedentary during a day, while prospective cohort studies identified sedentary behavior as an independent risk factor for NAFLD development and potentially progression (32, 33). A large prospective randomized Da Quing study including 110,660 men and women with glucose impairment showed exercise was associated with 46% (*P* < 0.0005) reduction of risk in developing diabetes, irrespective of baseline glucose levels and body mass index (BMI), suggesting a vital role of physical activity in the prevention of metabolic disorders associated with insulin resistance (34). The results of the HELENA study suggest that high cardiorespiratory fitness (CRF) might have protective effects on liver enzyme levels in adolescents with high waist circumference and that the exercise focusing on increasing CRF and decreasing abdominal fat might be a good tool in the prevention and treatment of NAFLD during adolescence (35). A study by Sung and co-workers following 169,347 men and women by ultrasound for 5 years provided the first longitudinal epidemiological data supporting the role of exercise in both the prevention and treatment of NAFLD. During follow-up, out of 126,811 adults without NAFLD at baseline, 23% developed NAFLD at follow-up. On the other hand, of the 42,536 individuals with NAFLD at baseline, 34% of cases resolved. After adjusting for potential confounders, any moderate to vigorous exercise level was associated with a reduced risk of new NAFLD and resolution of already present NAFLD. The most significant benefits were seen while exercising 5 days per week and in the case of increasing the frequency of exercise bouts over time (36). Similar results were confirmed by another more recent longitudinal follow-up study where people who were already active or became physically active during the course of follow-up were less likely to develop NAFLD compared with those that remained inactive (OR = 0.75, *p* = 0.03 and 0.75, *p* = 0.04, respectively), irrespective of BMI (37).

## The Role of Physical Activity in the Nafld Treatment

**Figure 1:**
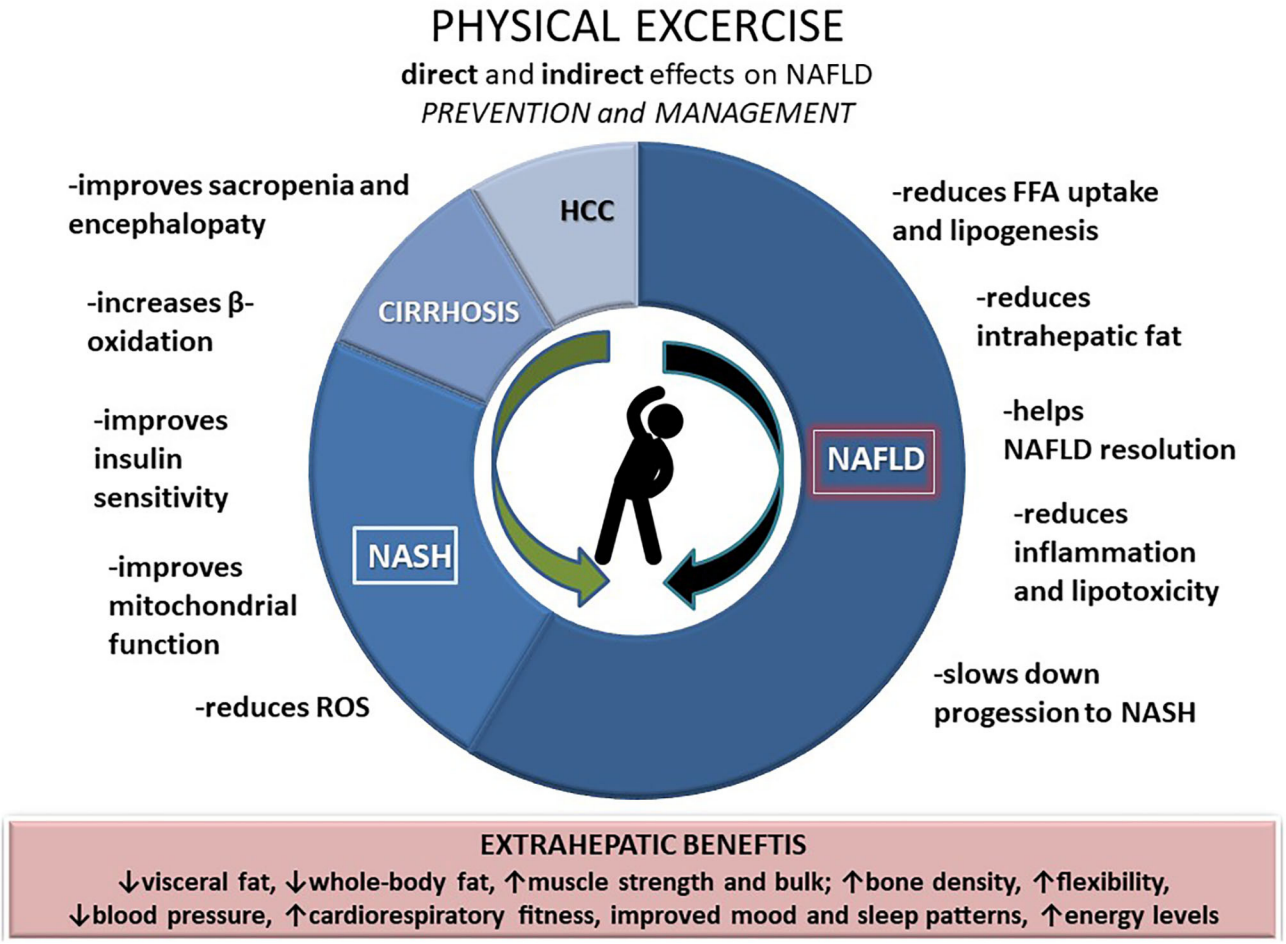
Direct and indirect effects of physical exercise on NAFLD.

Growing evidence highlights the need for physical activity in reducing the body weight (at best >10%) in order to improve liver histology and reduce fibrosis in NAFLD patients (38, 39). Weight loss achieved through physical activity improves hepatic and peripheral insulin sensitivity, but physical activity, regardless of the effects on body mass, also directly decreases the pro-inflammatory and oxidative stress markers and improves liver enzymes. According to the data from a recently published systemic review encompassing 24 exercise-only studies in NAFLD, structured exercise leads to a 20–30% relative reduction in hepatic steatosis, independent of weight loss (40). In addition, exercise might also affect the gut microbiota and modulate the liver inflammatory response and NASH progression (41) (Figure 1).

There is currently a gap in knowledge of the type, duration, and/or intensity of physical activity that would bring the best results for patients with NAFLD. It seems that both aerobic and anaerobic training for at least 4 months decrease to the same extent the overall adipose tissue, hepatic fat, and BMI, while no data exists on their potentially differential effects on liver histology (22). On the other hand, liver histology tends to depend on the exercise intensity and, according to some data, improves more with high-intensity activity (22, 42).

## Extrahepatic Benefits of Physical Activity and how it Affects Nafld Prognosis

In a randomized control trial recruiting NAFLD patients, exercise was associated with improvement of endothelial function, evaluated by flow-mediated dilatation of the brachial artery (43). Mentioned NO-dependent vascular dilatation is an important protective mechanism for cardiovascular health. Moreover, with its effect on muscle mass, physical activity reduces the risk of sarcopenia and improves cardiorespiratory fitness, which is low, especially in patients with advanced liver disease such as cirrhosis (19). Physical activity improves insulin sensitivity on the peripheral tissues and the liver and improves glucose metabolism (or glycemic control in clinically manifest diabetes), slowing down NAFLD progression and reducing overall cardiovascular risk. Moreover, it reduces systemic inflammation, lowers arterial blood pressure, and improves dyslipidemia (44). The established beneficial effect of physical activity on cardiovascular health reported in the general population is also applicable for the NAFLD patients, and given that cardiovascular disease remains the leading cause of death in NAFLD patients, encouraging regular exercise should be advocated and prescribed to all NAFLD patients, and during the entire disease course (45).

## Recommendations for Physical Activity in the Nafld Patients

Physical activity and specially structured exercises offer benefits independent of weight loss and represent the core treatments for NAFLD patients. Both aerobic and resistance training effectively reduce hepatic steatosis and reduce the NAFLD-associated cardiovascular risk (46). The exercise program should be tailored to a patient's preference and capacity, depending on physical fitness level, stage of the liver disease, and other comorbidities. High-intensity interval training (HIIT) is an attractive exercise modality for treating patients with NAFLD, especially those who lack time to exercise, while it reduces visceral adipose tissue, intrahepatic fat, and fibrosis (47). General recommendations include 150 min of weekly accumulated moderate-intensity aerobic exercise, accompanied by strength and endurance training at least two to three times weekly, avoiding consecutive days and including 8–10 exercises using the major muscle groups, with 10–15 repetitions in a moderate to high intensity. In addition, just reducing or breaking up sedentary time by few minutes of walking should also be a therapeutic target for patients who cannot attend the structured exercise programs. To assure the therapeutic effects, attention should be paid to patients' compliance to exercise and attain exercise goals (48, 49).

As majority of NAFLD patients are obese, special attention must be put on an exercise program which would be doable and also lead to a meaningful weight loss (10%) and improvements in cardiorespiratory fitness to provide health benefits (39). Current literature supports the evidence that both aerobic and anaerobic exercise with a duration of 20–60 min per session when performed in moderate intensity and practiced 4–7 days weekly for at least 6 months (with and without diet restriction) can lead to improvements in liver histology and therefore reversal of liver damage in NASH patients (50), while recently published study on overweight and obese patients supports the beneficial role of aerobic exercise regardless of dose and intensity (low-intensity/ high-volume, high-intensity/low-volume, low-intensity/low-volume) on the reduction of liver fat content (42). Therefore, recent guidelines emphasize the importance of exercise but leave the choice of training to be individually tailored according to patients' preferences and likelihood of adherence to exercise program in the long term (51).

## Author Contributions

MC: drafted and wrote and reviewed the manuscript. IB-C and AM: collected data and wrote and reviewed the manuscript. VC: critically reviewed the manuscript. All authors contributed to the article and approved the submitted version.

## Conflict of Interest

The authors declare that the research was conducted in the absence of any commercial or financial relationships that could be construed as a potential conflict of interest.

## Publisher's Note

All claims expressed in this article are solely those of the authors and do not necessarily represent those of their affiliated organizations, or those of the publisher, the editors and the reviewers. Any product that may be evaluated in this article, or claim that may be made by its manufacturer, is not guaranteed or endorsed by the publisher.
